# Hypersensitivity to liraglutide: A case report

**DOI:** 10.5415/apallergy.0000000000000149

**Published:** 2024-06-14

**Authors:** Ana G. Marino-Fernández, Irene García-Gutiérrez, Sofía Alonso Juaristi, Jaime López Gutiérrez, Mariam Tawfiq Piedad, Ángel L. Guerrero Sotelo, Ángel J. Albarracín Contreras, Pilar Ortiz Aljaro, Fernando Rodríguez Fernández

**Affiliations:** Allergology Service, Hospital Universitario Marqués de Valdecilla, Santander, Spain

**Keywords:** Hypersensitivity, immediate reaction, individualized management, liraglutide

## Abstract

Saxenda (liraglutide) is approved for the treatment of type 2 diabetes mellitus and chronic weight management in adults with obesity. We present the case of a 44-year-old woman with grade 2 obesity who was treated with liraglutide for weight reduction, initially well-tolerated. Despite a brief pause in liraglutide treatment for 5 days due to hospitalization, she developed, after the reintroduction of this treatment, a localized reaction characterized by maculopapular erythema and pruritus at the injection site. Skin tests revealed an acute positive reaction to liraglutide. Literature suggests variability in allergic reactions among glucagon-like peptide-1 receptor agonists, highlighting the need for further research. Our case underscores the importance of individualized management in treating adverse reactions to liraglutide in obese patients without type 2 diabetes mellitus.

## 1. Introduction

Saxenda (liraglutide) is a glucagon-like peptide-1 receptor agonist (GLP-1RA). It is approved by the US Food and Drug Administration and the European Medicines Agency (EMA) for the treatment of type 2 diabetes mellitus (T2DM) and chronic weight management in adults with a body mass index (BMI) of 30 kg/m^2^ or higher, or a BMI of 27 kg/m^2^ or higher with at least one weight-related comorbidity. It is administered subcutaneously once daily. The adverse effects of liraglutide primarily encompass gastrointestinal symptoms; moreover, it can also cause headache, insomnia, asthenia, and allergic reactions [1].

While there are currently other GLP-1RAs approved by the EMA for the treatment of T2DM, none of them are also approved for the treatment of weight management in adults, and only Saxenda has been approved for both indications.

## 2. Case report

A 44-year-old woman diagnosed with grade 2 obesity and vertical gastrectomy in December 2021, without previous history of any allergies, initiated a treatment for weight reduction with Saxenda 6 mg/mL (liraglutide) subcutaneously, 1.2 mg once daily. After 11 days of treatment, she paused it for 5 days due to hospitalization for pneumonia and reintroduced the treatment with the same regimen. After 7 days, she began experiencing a large local reaction consisting of maculopapular erythema with pruritus around the medication injection site, without reaction at another level, occurring approximately 10 minutes after subcutaneous injection. While she continued with the treatment (8 days more), the dermal reaction persisted, and it became more extensive, spreading across the abdomen. After 1 week of discontinuing the treatment, the local reaction resolved completely without any antiallergic treatment. There was no systemic reaction at any point despite continued treatment those days.

**Figure 1.**
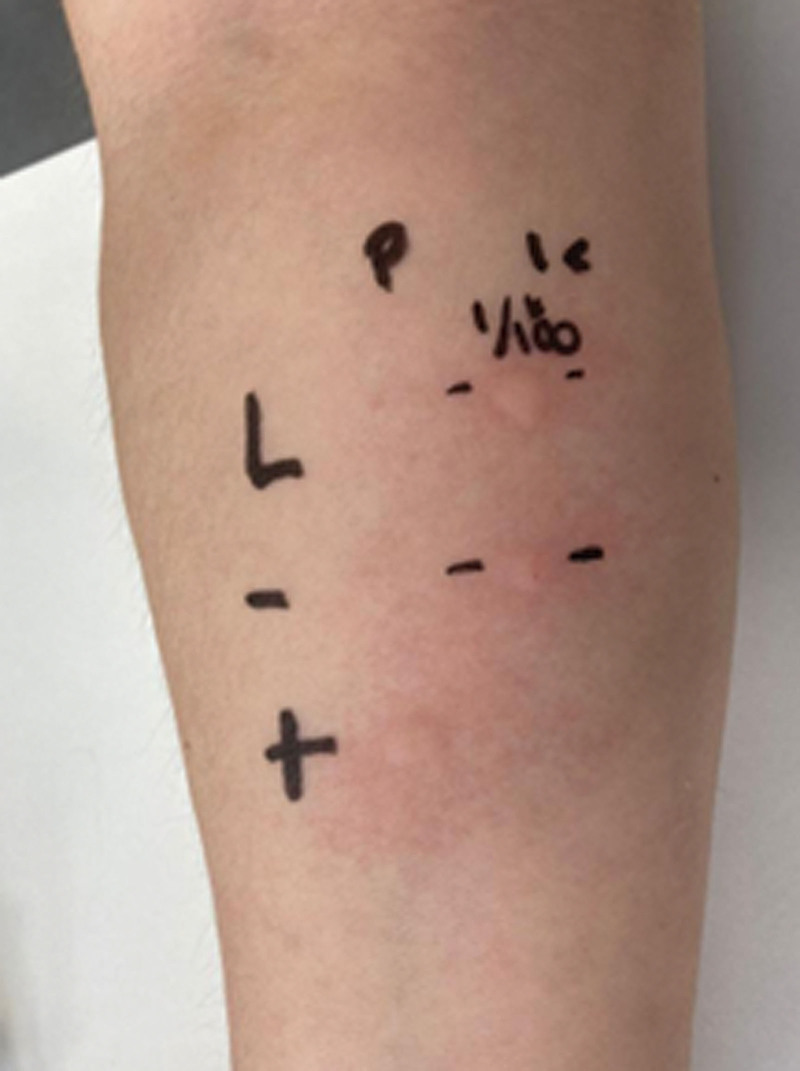
Negative intraepidermal skin test (P) for liraglutide with a positive control (+) using histamine (7 × 5 mm) and a negative control (−) using physiological saline solution (0 × 0 mm). Positive intradermal skin test (IC) for liraglutide 1/100 at 8 × 8 mm with a negative control (−) using physiological saline solution (0 × 0 mm).

After informed consent was signed, several cutaneous tests were performed, starting with an intraepidermal test with liraglutide 1/1 that yielded negative results. Subsequently, an intradermal test at a concentration of 1/100 was performed [2], resulting in an acute positive reaction to liraglutide at 8 × 8 mm (see Fig. 1). The skin test had a positive histamine control and a physiological saline solution negative control [2]. The positive dilution (1/100) was tested in 5 healthy control individuals who had not been exposed to the drug, with negative results at the immediate and delayed readings.

Then, the case was discussed with endocrinology. Since liraglutide is the only GLP-1RA approved by the EMA for obesity, we offered the possibility of performing desensitization; however, they decided to change the therapeutic approach to diet and exercise. Therefore, liraglutide was discontinued and it was determined that its administration should be avoided.

## 3. Discussion

Liraglutide, as a GLP-1RA, aids in weight management by regulating appetite. It induces feelings of satiety and fullness and improves glycemic control when combined with diet and exercise. It has become a valuable tool in preventing obesity-related complications [3]. There are other GLP-1RAs that present differences in their components. Lixisenatide and exenatide are exendin-4-based GLP-1RA, they are homologous except for the N-terminal. Liraglutide and dulaglutide are an analogy to human GLP-1RA and appear to be less immunogenic than exenatide and lixisenatide. Semaglutide is another analogy to human GLP-1RA derived from liraglutide, but there is a change in its molecular components.

Literature reports have described cases of allergy reactions to GLP-1RA. Most of these reports indicate nonimmediate allergic reactions to GLP-1RA. These adverse reactions include local cutaneous reactions such as maculopapular erythema, generalized cutaneous reactions such as acute photodistributed generalized exanthematous pustulosis, urticaria or maculopapular rash, and anaphylaxis.

Carvallo et al. [2] presented a case of liraglutide hypersensitivity with delayed positive intradermal tests after 24 hours; the negative skin tests for semaglutide suggested it could be used as an alternative.

Shamriz et al. reported a case of anaphylaxis to lixisenatide. The patient initiated treatment for T2DM with exenatide, which was replaced by liraglutide, and after 2 years it was modified to lixisenatide; the patient experienced an anaphylactic shock after administration of lixisenatide. They conducted skin tests: intraepidermal tests with exenatide were positive, intradermal tests with lixisenatide were positive; while both intraepidermal and intradermal tests with liraglutide were negative. The decision was made to resume treatment with liraglutide, which the patient tolerated [4].

Cogen et al. [5] documented a case of acute photodistributed generalized exanthematous pustulosis associated with liraglutide; although no allergological skin tests were conducted, histological examination and the patient’s blood test results were compatible with the diagnosis. Another case of vesiculopustular dermatosis associated with liraglutide was described by Besemer et al. [6], supported by compatible skin biopsy findings. Neel et al. [7] described a liraglutide-induced injection site but did not show a reaction in skin tests or biopsy, it resolved upon discontinuation of treatment. Bovijn et al. [8] reported a case of generalized cutaneous reaction to liraglutide, with peripheral eosinophilia; they conducted a skin biopsy but did not perform skin tests with the drug. Ouellette et al. described 2 reports of hypersensitivity to semaglutide; the first one presented a case with multiple erythematous plaques and was supported by compatible biopsy and laboratory findings, no allergological tests were performed; the second case was presented with pruritic maculopapular erythema, with negative patch testing and resolution of symptoms after discontinuation of semaglutide [9].

Yeğit et al. [10] presented 2 cases of allergy to exenatide and an effective protocol of desensitization to this drug, highlighting the importance of desensitization for those patients without available alternative therapies.

In the case of our patient, liraglutide was administered not for T2DM but for overweight, and unlike the previously cited authors, the intradermal test with acute positive reaction suggests an immediate sensitization to liraglutide.

In the last few years GLP-1RA allergy reactions have been described, there are some studies that suggest the possibility of conducting desensitization to GLP-1RAs, whereas others suggest that due to the chemical structure of GLP-1RAs, there might be a chance of no cross-reactivity among them. It would be advisable to conduct further research to establish a clear pattern. In our specific case, the patient did not fulfill the desensitization criteria and no other GLP-1RAs were tested because there were no other alternatives approved at that moment.

The cutaneous reaction in our patient was resolved by avoiding the drug. That is why it is important to emphasize the significance of individualized management in treating adverse reactions to liraglutide in patients like ours, who are obese and without T2DM.

## Conflicts of interest

The authors declare no conflicts of interest.

## Author contributions

Ana G. Marino-Fernández: Conception and design of the work; analysis and interpretation of data for the work; and drafting the work and revising it critically. Irene García-Gutiérrez: Design of the work; acquisition, analysis, and interpretation of data for the work; and revising it critically. Sofía Alonso Juaristi: Acquisition of data for the work and revising it. Jaime López Gutiérrez, Mariam Tawfiq Piedad, Ángel L. Guerrero Sotelo, Ángel J. Albarracín Contreras, and Pilar Ortiz Aljaro: Design of the work and revising it. Fernando Rodríguez Fernández: Design of the work; analysis and interpretation of data for the work; and revising it critically. All authors approved the final version of the article.
